# In patients supported with peripheral veno-arterial extracorporeal membrane oxygenation, what factors are associated with the development of spinal cord ischaemia?

**DOI:** 10.1093/icvts/ivae052

**Published:** 2024-03-26

**Authors:** Alison Zhu, Charis Tan, Richard Chard, Yishay Orr

**Affiliations:** Department of Cardiothoracic Surgery, Westmead Hospital, Westmead, NSW, Australia; Discipline of Surgery, Faculty of Medicine and Health, University of Sydney, NSW, Australia; Department of Cardiothoracic Surgery, Westmead Hospital, Westmead, NSW, Australia; Discipline of Surgery, Faculty of Medicine and Health, University of Sydney, NSW, Australia; Department of Cardiothoracic Surgery, Westmead Hospital, Westmead, NSW, Australia; Discipline of Surgery, Faculty of Medicine and Health, University of Sydney, NSW, Australia; Department of Cardiothoracic Surgery, Westmead Hospital, Westmead, NSW, Australia; Discipline of Surgery, Faculty of Medicine and Health, University of Sydney, NSW, Australia

**Keywords:** Review, Extracorporeal membrane oxygenation, Spinal cord ischaemia, Paraplegia

## Abstract

A best evidence topic in cardiac surgery was written according to a structured protocol. The question addressed was ‘in patients supported with peripheral veno-arterial extracorporeal membrane oxygenation, what factors are associated with the development of spinal cord ischaemia’? Altogether, more than 22 papers were found using the reported search, of which 10 represented the best evidence to answer the clinical question. The authors, journal, date and country of publication, patient group studied, study type, relevant outcomes and results of these papers are tabulated. Of the 28 patients reported by included studies, the thoracic spinal cord was most commonly affected. Twenty patients (71%) survived to hospital discharge and 7 (25%) were reported to have neurological recovery. Potential confounders included coronary angiography, cardiac arrest requiring chest compressions and concomitant intra-aortic balloon pump. Consequently, all papers highlighted the likely multifactorial aetiology of spinal cord infarction in these patients. We propose that close neurological observation, particularly in patients who have received chest compressions, and management of potential aetiological factors is crucial to aid in timely diagnosis and potential prevention of this rare complication. Limiting sedation and neuromuscular blockade to enable neurologic assessment of the lower limbs may allow more timely diagnosis.

## INTRODUCTION

A best evidence topic was constructed according to a structured protocol. This is fully described in the *ICVTS* [1].

## THREE-PART QUESTION

In patients supported with peripheral veno-arterial extracorporeal membrane oxygenation (VA-ECMO), what factors are associated with the development of spinal cord ischaemia?

## CLINICAL SCENARIO

A 55-year-old patient underwent coronary artery bypass grafting and aortic valve replacement, requiring an intra-aortic balloon pump (IABP) to be weaned off cardiopulmonary bypass. Eight hours postoperatively, due to rising inotropic and vasopressor requirements, he was placed on peripheral VA-ECMO for low cardiac output state. After 7 days, he is weaned from VA-ECMO but is noted to have bilateral lower limb paralysis. Spinal magnetic resonance imaging (MRI) is consistent with spinal cord infarction (SCI). You wonder whether prolonged duration on peripheral VA-ECMO could have contributed to his spinal cord ischaemia. You resolve to check the literature for evidence.

## SEARCH STRATEGY

The literature search was performed in Medline from 1946 to July 2023 using the Ovid interface using the search terms: (Extracorporeal Membrane Oxygenation/OR extracorporeal membrane oxygenation.mp OR ecmo.mp) AND (Spinal Cord ischaemia/OR Spinal Cord Injuries/OR spinal cord infarct*.mp OR spinal cord ischaemia OR spinal cord ischaemia OR Paraplegia/OR paraplegi*.mp). A review of references of all papers was undertaken.

## SEARCH OUTCOME

Twenty-two papers were found using the reported search. An additional 2 papers were identified from review of references. From these, 10 papers were identified that provided the best evidence to answer the question. These are presented in Table 1.

**Table 1:** Best evidence papers

Author, date, journal and countrystudy type(level of evidence)	Patient group	Outcomes	Key results	Comments
Pasrija et al. (2023),J Cardiothorac Vasc Anesth, USA [2]Case series (level IV)	7 patients Male, 62 years Female, 44 years Male, 46 years Male, 34 years Male, 65 years Male, 60 years Female, 70 years 3 patients had concomitant IABP 4 patients underwent coronary angiography	Duration of VA-ECMO (days)	15 (10.5–18.5)	Only 4 patients had SCI vertebral level of infarction diagnosed on MRI. The remaining 3 were assessed clinically, with diagnoses of SCI from T10–12 to conus
		ECMO flow rate (l/min)	4.6 (4–5)	
		Days to first evidence of neurological deficit from initiation of VA-ECMO	7 (6–10)	
		Level of SCI on MRI (*n*=4)	L1 to conus T11 to conus T11 to conus T10 to conus	
		Hospital survival	4 (57%)	
		Neurological recovery	2 (29%)	
Le Guennec *et al* (2020),J Artif Organs, France [3]Case series (level IV)	6 patients Male, 35 years Male, 48 years Male, 56 years Male, 62 years Male, 43 years Male, 62 years 2 patients had concomitant IABP 4 patients underwent coronary angiography	Duration of VA-ECMO (days)	8.5 (4–25)	1 year follow-up duration 5 patients had been weaned off ECMO prior to development of neurological deficits Slow sedation wean and low index of clinical suspicion may have delayed diagnosis
		ECMO flow rate (L/min)	3.95 (3.83–4.08)	
		Days to 1st evidence of neurological deficit from initiation of VA-ECMO	9 (6.25–19.25)	
		Level of SCI on MRI	T1 to conus T9 to conus T10 to conus T6 to conus Conus T12 to conus	
		Hospital survival	5 (83%)	
		Neurological recovery	3 (50%)	
Salna *et al*. (2021),Ann Thorac Surg, USA [4]Case series (level IV)	4 patients Male, 29 years Female, 59 years Female, 46 years Female, 56 years No IABP 1 patient underwent coronary angiography	Duration of VA-ECMO (days)	11.5 (9–15.25) days	No MRI obtained for 1 patient Although flow rate target reported, it is unclear if this was met Hospital survival and neurological recovery not reported
		ECMO flow rate target (ml/kg/min)	>2.5	
		Days to 1st evidence of neurological deficit from initiation of VA-ECMO	13 (11–16.75)	
		Level of SCI on MRI (*n*=3)	T4 to conus L1/L2 infarct T5 to T10	
Gangahanumaiah *et al*. (2021),Eur Heart J, Case Rep, Australia [5]Case series (level IV)	3 patients Female, 49 years Male, 73 years Female, 38 years No IABP 1 patient underwent coronary angiography	Duration of VA-ECMO (days)	10 (10–11.5)	Although flow rate and MAP targets were reported, it is unclear if these were met Slow sedation wean may have delayed diagnosis
		ECMO flow rate target (l/min)	3.5-5	
		MAP target (mmHg)	>65	
		Days to 1st evidence of neurological deficit from initiation of VA-ECMO	15 (12.5–17)	
		Level of SCI on MRI	T2 to conus T6 to conus T9 to conus	
		Hospital survival	0 (0%)	
		Neurological recovery	0 (0%)	
Samadi *et al*. (2016),Crit Care Med, Australia [6]Case series (level IV)	3 patients Female, 37 years Female, 43 years Female, 19 years All patients had concomitant IABP No coronary angiography	Duration of VA-ECMO (days)	9 (6.0–9.5)	No VA-ECMO flow rates were reported Slow sedation wean may have delayed diagnosis
		Days to 1st evidence of neurological deficit from initiation of VA-ECMO	10 (8.5–10.0)	
		Level of SCI on MRI	T1 to conus Conus C5 to conus	
		Hospital survival	2 (66%)	
		Neurological recovery	1 (33%)	
		Days to first evidence of neurological deficit from initiation of VA-ECMO	1	
		Level of SCI on MRI	T5 to conus	
		Hospital survival	1 (100%)	
		Neurological recovery	0	
Itagaki *et al*. (2022),Medicine, Japan [7]Case report (level IV)	1 patient Male, 78 years Concomitant IABP Underwent coronary angiography	Duration of VA-ECMO (days)	3	Single patient experience Reduced level of consciousness may have delayed diagnosis
		Days to first evidence of neurological deficit from initiation of VA-ECMO	14	
		Level of SCI on MRI	T5 to T12	
		Hospital survival	1 (100%)	
		Neurological recovery	0 (0%)	
Chien *et al*. (2021),Interdiscip Neurosurg, Taiwan [8]Case report (level IV)	1 patient Male, 26 years No IABP No coronary angiography	Duration of VA-ECMO (days)	3	Single patient experience
		Days to 1st evidence of neurological deficit from initiation of VA-ECMO	NR	
		ECMO flow rate (l/min)	2.8	
		Level of SCI on MRI	T9 to L1	
		Hospital survival	1 (100%)	
		Neurological recovery	0 (0%)	
Shin *et al*. (2018),Acute Crit Care, Korea [9]Case report (level IV)	1 patient Female, 81 years No IABP Underwent coronary angiography	Duration of VA-ECMO (days)	NR	Single patient experience
		Days to 1st evidence of neurological deficit from initiation of VA-ECMO	1	
		Level of SCI on MRI	T5 to conus	
		Hospital survival	1 (100%)	
		Neurological recovery	0	
Magnusson *et al*. (2018),Clin Case Rep, Sweden [10]Case report (level IV)	1 patient Female, 28 years No IABP Underwent coronary angiography	Duration of VA-ECMO (days)	21	Single patient experience Slow sedation wean may have delayed diagnosis
		Days to 1st evidence of neurological deficit from initiation of VA-ECMO	21	
		Level of SCI on MRI	T6 to conus	
		Hospital survival	1 (100%)	
		Neurological recovery	0	
Oda *et al*. (2010),J Artif Organs, Japan [11]case report (level IV)	1 patient Male, 6 years No IABP No coronary angiography	Duration of VA-ECMO (days)	4	Single patient experience Slow sedation wean may have delayed diagnosis
		Days to first evidence of neurological deficit from initiation of VA-ECMO	6	
		Level of SCI on MRI	T4-5	
		Hospital survival	1 (100%)	
		Neurological recovery	0	

Values presented as median (IQR) or *n* (%).

IABP: intra-aortic balloon pump; MRI: magnetic resonance imaging; NR: not reported; SCI: spinal cord infarction; VA-ECMO: veno-arterial extracorporeal membrane oxygenation.

## RESULTS

Pasrija *et al.* [2] report a case series of 7 patients who developed SCI while supported on peripheral femoral VA-ECMO. The median duration of VA-ECMO was 15 (10.5–18.5) days. The median time to 1st neurological evidence of SCI was 7 (6–10) days. While all patients were reported to have SCI involving the lower thoracic spinal cord to cauda equina, 3 of these patients were diagnosed solely through clinical assessment due to MRI incompatibility with implanted ECMO devices and did not have imaging confirmation. Four patients (57%) survived the hospital stay with 2 of these patients demonstrating neurological recovery. The authors postulate that SCI developed due to spinal cord oedema from hyperperfusion or venous congestion.

Le Guennec *et al.* [3] present 6 patients who developed SCI after circulatory support with peripheral VA-ECMO. Median duration of ECMO was 8.5 (4–25) days. After development of neurological symptoms, spinal MRI demonstrated spinal cord infarction from thoracic spinal cord to conus in 5 patients. One patient had spinal cord infarction involving only conus. Five patients survived to hospital discharge, and 1 patient developed ventilator-associated pneumonia and septic shock, succumbing to refractory multiorgan failure. All patients who survived were followed-up after 1 year, with neurological recovery in 3 patients. This study is limited by confounding variables, including cardiac arrest, concomitant IABP and coronary angiography, which may contribute to SCI development due to embolic or hypoperfusion phenomena. Only 1 patient reported neurological deficits while on ECMO, while the remaining patients had already been weaned off ECMO. Delayed diagnosis resulted from limited neurological assessment during sedation and initial attribution of deficits to critical illness myopathy. The authors conclude that there is likely multifactorial aetiology for development of SCI, including spinal artery microembolism from cannulas associated with VA-ECMO, IABP or coronary angiography, prolonged spinal cord hypoperfusion and vasopressor use.

Salna *et al.* [4] present 4 patients who developed SCI after being supported on peripheral VA-ECMO. Median duration of VA-ECMO prior to diagnosis of SCI was 11.5 (9–15.25) days. One patient developed SCI while on femoral ECMO, while 2 patients had already been weaned off femoral ECMO. The last patient was converted from common femoral VA-ECMO to right internal jugular vein-axillary artery ECMO 12 days after initiation of support to facilitate mobility. After the conversion of configuration and subsequent cessation of sedation, the patient was noted to have bilateral paralysis and areflexia. SCI was diagnosed clinically as patient instability prevented MRI. The remaining 3 patients had evidence of infarction involving the thoracic spinal cord on MRI. A 25-day delay of SCI diagnosis occurred in 1 patient, where the initial assessment of weakness was attributed to critical illness neuropathy. The authors raise concerns that turbulent flow at the point at which blood flow from native cardiac output meets retrograde femoral flow from VA-ECMO in the descending thoracic aorta may result in thrombosis, contributing to SCI.

Gangahanumaiah *et al.* [5] present a case series of 3 patients who were supported with peripheral VA-ECMO for cardiogenic shock and subsequently developed SCI. Duration of VA-ECMO was a median of 10 (10–11.5) days. All patients developed bilateral lower limb paralysis with a median onset of 15 (12.5–17) days. One patient was diagnosed with SCI on the day of ECMO decannulation. Two patients were not weaned off sedation for a further 5 and 7 days, respectively, thus delaying diagnosis of paraplegia and subsequent MRI confirming SCI. Spinal MRI revealed spinal cord infarction involving the thoracic spinal cord to conus in all 3 patients. All patients developed multiorgan failure and sepsis and did not survive their hospital admission. The authors postulate relative spinal cord hypoperfusion caused by watershed phenomenon in peripheral VA-ECMO whereby oxygenated blood from the femoral outflow cannula competes with native antegrade cardiac output, resulting in a potential area of low flow in the aorta, giving rise to relative ischaemia and infarction.

Samadi *et al.* [6] report a case series of 3 patients who were supported on VA-ECMO combined with IABP for viral cardiomyopathy, peripartum cardiomyopathy and cardiogenic shock after acute myocardial infarction, and subsequently developed SCI. Duration of VA-ECMO was a median of 10 (8.5–10.0) days. One patient developed SCI symptoms while on ECMO, whereas 2 patients had delay in diagnosis due to slow sedation wean. Spinal MRI revealed SCI involving the thoracic spinal cord to conus in all patients. Two patients survived hospital discharge with only 1 demonstrating neurological recovery. The authors suggest small aortic calibre, loss of native ejection and shock states requiring high-dose vasopressor support as risk factors for the development of SCI in patients on ECMO and IABP.

Itagaki *et al.* [7] report a case of a 78-year-old male who was placed on peripheral VA-ECMO for cardiogenic shock after an acute myocardial infarction. Notably, he sustained 2 cardiac arrests with total downtime of 13 min. He underwent emergency coronary angiography after initiation of IABP and VA-ECMO support. His duration of VA-ECMO was 3 days; however, he only regained consciousness on day 12 and was extubated on day 14, when the authors report the initial observation of lower limb neurological deficit. Spinal MRI revealed SCI from T5 to T12. This patient survived his hospital admission but demonstrated no neurological recovery. The authors conclude that while SCI after VA-ECMO is rare, regular neurological examination and maintaining adequate blood pressure may assist in early detection and prevention of this complication, respectively.

Chien *et al.* [8] report the case of a 26-year-old male who was placed on peripheral VA-ECMO with subsequent development of SCI. The patient was placed on femoral VA-ECMO due to haemodynamic instability from viral cardiomyopathy secondary to influenza A pneumonia with an initial flow rate of 2.8 l/min. Total duration of ECMO was 4 days, with findings of progressive lower limb weakness from day 3. MRI demonstrated SCI from T9 to L1. The patient survived to hospital discharge with persistent paraplegia and lower limb hypoesthesia. The authors suggest a multifactorial aetiology of SCI in this case, including ECMO, profound hypotension and inotrope-related vasospasm.

Shin *et al.* [9] report the case of an 81-year-old female who required support on peripheral VA-ECMO due to cardiogenic shock following acute myocardial infarction. Neurological deficit was noticed just 1 day after VA-ECMO initiation. Due to persistence of bilateral lower limb weakness after removal of VA-ECMO, spinal MRI was undertaken on day 5 that revealed SCI involving T5 to conus. The patient survived the hospital admission but did not demonstrate neurological recovery with persisting distal lower limb weakness. The authors observe that neurology is commonly intact prior to initiation of ECMO, suggesting a correlation between period of circulatory support and SCI. The authors suggest maintaining a high clinical suspicion for SCI in the event of lower limb weakness in patients on VA-ECMO to aid in timely diagnosis and institution of supportive measures.

Magnusson *et al.* [10] report the case of a 28-year-old female who required VA-ECMO for fulminant peripartum myocarditis presenting with cardiac arrest requiring mechanical cardiopulmonary resuscitation. She remained on VA-ECMO for 21 days, with neurological deficit 1st noted following weaning from ECMO and sedation. Spinal MRI revealed SCI involving T6 to conus. The patient survived the hospital admission but did not demonstrate neurological recovery with persisting paraplegia. The authors suggest contributors to SCI as spinal hypoperfusion in the setting of hypotension and/or vasopressor use and possible spinal artery embolism arising from the VA-ECMO circuit.

Oda *et al.* [11] report the case of a 6-year-old male who was initially placed on peripheral venovenous-ECMO for Influenza A viral myocarditis that was complicated by persistent ventricular fibrillation. During 141 min of chest compressions, the ECMO configuration was altered to VA-ECMO by suturing a sidegraft to the left common femoral artery. After 4 days, he was converted back to venovenous-ECMO for another 2 days of support, was then weaned off ECMO and successfully extubated the following day. Three days after initial weaning of VA-ECMO, the patient was noted to have paraplegia and bowel and urinary incontinence. Spinal MRI revealed SCI involving T4 to T5. The patient survived the hospital admission but did not demonstrate neurological recovery with persisting paraplegia. The authors suggest SCI arising from hypotension during prolonged chest compressions resulting in spinal cord hypoperfusion. They conclude that immediate institution of VA-ECMO may have helped avoid SCI.

## CLINICAL BOTTOM LINE

Spinal cord ischaemia is a rare potential complication of peripheral VA-ECMO, with an estimated overall incidence between 0.3% and 0.6% for patients supported with peripheral VA-ECMO [3, 5]. Pasrija *et al.* report an incidence of 3% in patients on femoral VA-ECMO, suggesting femoral cannulation may be associated with development of SCI [2]. We summarize 10 studies reporting 28 patients who developed SCI after peripheral VA-ECMO support, of which the thoracic spinal cord was most commonly affected. Twenty patients (71%) survived to hospital discharge and 7 (25%) were reported to have neurological recovery. Unfortunately, no control group exists and the denominator of total VA-ECMO cases is unknown, so the exact incidence of SCI remains undefined. Potential confounders included 12 patients who underwent coronary angiography, 11 patients who experienced cardiac arrest requiring cardiopulmonary resuscitation with chest compressions and 9 patients who had concomitant IABP. Consequently, all papers highlighted the likely multifactorial aetiology of SCI in these patients. Hypotheses include microembolism and thrombosis from turbulent flow of the ECMO circuit, spinal cord hypoperfusion from watershed phenomenon, hypoperfusion during cardiopulmonary resuscitation, spinal cord oedema and vasopressor use. This review is limited both by the small sample sizes and nature of VA-ECMO, with many patients requiring sedation and critical organ support, thus confounding association and delaying diagnosis. Notably, lower limb weakness was often initially attributed to critical illness neuropathy, delaying spinal cord imaging and institution of supportive care. We propose that close neurological observation, particularly in patients who have received chest compressions, and management of potential aetiological factors [e.g. maintaining adequate mean arterial blood pressure, anticoagulation and avoiding IABP (if possible)] is crucial in patients supported with peripheral VA-ECMO to aid in timely diagnosis and potential prevention of this rare complication. Limiting sedation and neuromuscular blockade to enable neurologic assessment of the lower limbs may allow more timely diagnosis. Further studies to elucidate potential pathologic mechanisms will be essential to mitigating this unfortunate outcome.

## Data Availability

Data availability is not applicable to this article as no new data were created or analysed in this study.
